# How the heart team is portrayed in online news

**DOI:** 10.1016/j.xjon.2026.101803

**Published:** 2026-04-08

**Authors:** Joshua R. Chen, Vishal N. Shah, Purab D. Kothari, Adam M. Ostrovsky, Joseph E. Bavaria

**Affiliations:** Department of Cardiothoracic Surgery, Thomas Jefferson University Hospital, Philadelphia, Pa

**Keywords:** heart team, cardiologists, cardiothoracic surgeons, online health, cardiovascular news ecosystems

## Abstract

**Background:**

Online news plays a critical role in public health literacy and the dissemination of medical information. As cardiovascular care increasingly relies on multidisciplinary collaboration, understanding how different specialties are represented in public portrayals of the heart team is essential; accordingly, this study examines patterns in online cardiovascular news coverage to assess how these roles are depicted.

**Methods:**

A systematic Google News search was conducted using 7 keywords: “heart attack,” “coronary artery disease,” “heart failure,” “aortic stenosis,” “aortic regurgitation,” “mitral stenosis,” and “mitral regurgitation.” Articles published between January 1, 2023, and January 1, 2024, were screened. Press type and physician specialty, gender, and role were analyzed.

**Results:**

Of the 1580 articles screened, 1131 met our inclusion criteria for analysis. Cardiologists accounted for more than one-half of all featured specialist appearances (640 of 1200; 53.3%), followed by interventional cardiologists (277 of 1200; 23.1%), other physicians (165 of 1200; 13.8%), and cardiothoracic surgeons (118 of 1200; 9.8%). Cardiothoracic surgeons were least represented in coverage of heart attack, coronary artery disease, and heart failure (2.4%, 3.0%, and 2.9%, respectively) but appeared more frequently in articles focused on valvular disease. Most physicians were academically affiliated and appeared mainly as interviewees. Female physicians were underrepresented overall.

**Conclusions:**

Online cardiovascular news tends to feature cardiologists more often than cardiothoracic surgeons, emphasizing the importance of understanding how cardiovascular medicine is conveyed to the public.

**Figure:**
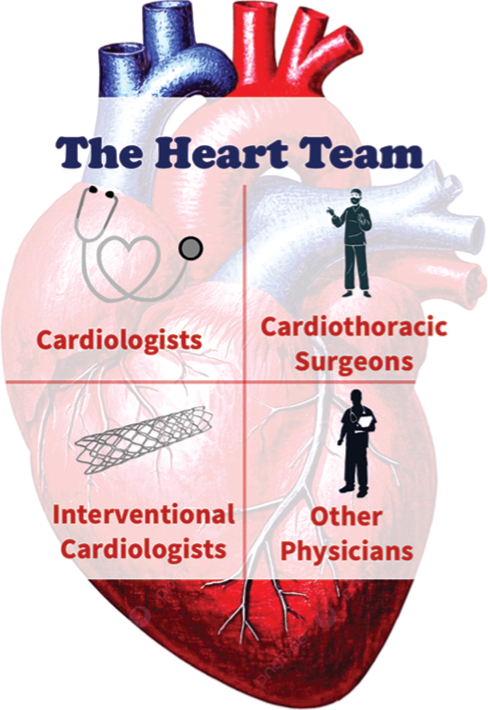
Physician representation in online cardiovascular news coverage.

**Central Message**

Online coverage of common cardiovascular diseases shows greater representation of cardiologists, with cardiothoracic surgeons featured relatively less frequently, except in valvular disease contexts.

**Perspective**

Exploring physician demographics and representation in online coverage of common cardiovascular diseases can help inform the multidisciplinary care model.

Online news media has become an influential component of the patient–provider relationship. Patients increasingly rely on news outlets to supplement their understanding of medical conditions and to learn about treatment options. At the same time, online news coverage shapes public perceptions of the healthcare system and medical specialties, often invoking physicians’ authority to confer credibility regarding medical content.^1^ Because physicians who contribute online news can shape these narratives, their participation influences both the accuracy of disseminated information and the public image of their specialties. As cardiovascular care becomes more multidisciplinary, understanding the representation of heart team members and other relevant physicians in public-facing media is increasingly important.^2^, ^3^, ^4^

Previous research in vascular disease has examined specialty-specific representation in online news, whereas studies in cardiovascular disease have focused primarily on social media engagement.^5^^,^^6^ No studies to date have examined how members of the heart team—including core members such as cardiologists and cardiothoracic surgeons, as well as other key noncore physicians (eg, emergency medicine physicians)—are represented in online news coverage of common cardiovascular diseases. Furthermore, the demographic characteristics of physicians featured in such content remain undefined.

To address this knowledge gap, we systematically examined how cardiologists, interventional cardiologists, cardiothoracic surgeons, and other physicians are represented in Google News articles related to common cardiovascular diseases. We aimed to quantify specialty representation and characterize the demographic and role-related attributes of physicians featured in online cardiovascular news ecosystems, allowing comparisons across specialties in the public portrayal of the heart team and other key physicians throughout the continuum of cardiovascular care.

## Methods

An online search was conducted using 7 diagnostic keyword phrases related to common cardiovascular diseases: “heart attack,” “coronary artery disease,” “heart failure,” “aortic stenosis,” “aortic regurgitation,” “mitral stenosis,” and “mitral regurgitation.” These keywords were selected by author consensus based on diseases managed in a multidisciplinary setting. Keywords specific to procedures—such as transcatheter aortic valve replacement (TAVR) and coronary artery bypass grafting—were excluded to minimize procedural bias, although this may have limited the visibility of cardiothoracic surgeons. This study used publicly available data, was exempt from institutional review, and did not require patient consent.

### Reproducibility Controls and Search Protocol

To enhance reproducibility, all searches were performed through Google News (news.google.com, US edition) rather than relying on general Google Search results. Searches were conducted on April 2, 2024, between 12:00 and 15:00 Eastern Standard Time in Philadelphia, Pennsylvania. Incognito mode was used to minimize algorithmic personalization, and all results were retrieved through the Google News tab.

Search results were limited to articles published between January 1, 2023, and January 1, 2024, establishing a clearly defined timeframe rather than attempting to infer temporal trends. For each keyword, the first 300 Google News results, sorted by “relevance”—a criterion influenced by search engine optimization, institutional press activity, and digital publishing practices—were collected, reflecting Google's retrieval limit. Exclusion criteria included subscription-only content, peer-reviewed scientific publications, and duplicate articles.

### Screening Process

Three authors (J.R.C., P.D.K., and A.M.O.) independently screened the articles. Duplicate or syndicated articles were screened for overlap to avoid redundant entries within each keyword. Each article was coded for press type, physician role, and the specialty of quoted physicians. Physicians mentioned but not interviewed, cited, or otherwise contributing were excluded.

### Press Type Classification

Medical press was defined as news articles published by healthcare organizations or medical media companies. Industry press was defined as articles published by pharmaceutical or medical device companies. Lay press was defined as general or mainstream news media outlets that did not meet the aforementioned criteria.

### Physician Demographics: Identification, Verification, and Definitions

Physician demographic information (eg, gender, specialty, academic rank, practice type) was collected through triangulation across verified online sources, including institutional and practice websites, as well as professional directories. When primary sources were incomplete, secondary commercial sites (eg, US News, Zocdoc, Healthgrades, Doximity, LinkedIn) were used.

Other physicians included those in family medicine and internal medicine, as well as specialists in emergency medicine, neurology, and endocrinology. Although not core members of the heart team, these physicians have integral roles in shaping referrals, managing key comorbidities, and supporting patient understanding of heart team decisions. Academically affiliated physicians are affiliated with universities, teaching hospitals, or academic medical centers, whereas non–academically affiliated physicians work in community hospitals, private practices, or other nonteaching clinical settings.

### Statistical Analysis

Descriptive statistics were generated. Formal hypothesis testing was deferred owing to variability across specialties and categories (eg, keywords, publication types), as well as potential sampling bias, to avoid overgeneralization or unsupported causal inferences. The primary aim was to characterize patterns, distributions, and frequencies. Keywords were visualized using bar graphs, and statistical analyses and data visualizations were performed using Microsoft Excel.

## Results

### Press Type Representation

A total of 1580 articles were screened, of which 1131 (71.6%) remained after application of exclusion criteria. Among the included articles, 854 (75.5%) were classified as medical press, 270 (23.9%) as lay press, and 7 (0.6%) as industry press.

Keyword-specific results demonstrated variability across press types and specialties. “Heart attack,” “coronary artery disease,” and “heart failure” each reached the maximum limit of 300 articles per Google search, whereas “mitral stenosis” yielded the fewest, with 71 articles. Among all keywords, “coronary artery disease” had the highest proportion of articles classified as medical press (173 of 207; 83.6%). “Aortic stenosis” had the highest representation in industry press (4 of 185; 2.2%) and “mitral stenosis” had the highest representation in the lay press (18 of 37; 48.6%). The results are presented in Table 1.

**Table 1:** Article characteristics

Keyword	Total articles	Relevant	Medical press	Lay press	Industry press
Heart attack	300	280 (93.3)	220 (78.6)	60 (21.4)	0 (0)
Coronary artery disease	300	207 (69.0)	173 (83.6)	33 (15.9)	1 (0.05)
Heart failure	300	237 (79.0)	182 (76.8)	55 (23.2)	0 (0)
Aortic stenosis	279	185 (66.3)	125 (67.6)	56 (30.3)	4 (2.2)
Aortic regurgitation	107	54 (50.5)	45 (83.3)	9 (16.7)	0 (0)
Mitral stenosis	71	37 (52.1)	19 (51.4)	18 (48.6)	0 (0)
Mitral regurgitation	223	131 (58.7)	90 (68.7)	39 (29.8)	2 (1.5)
Total	1580	1131 (71.6)	854 (75.5)	270 (23.9)	7 (0.6)

Categorical data presented as n (%).

Cardiologist involvement rates were similar between medical and lay press articles (488 of 854; 57.1% vs 151 of 270; 55.9%), as was the case for interventional cardiologists (202 of 854; 23.7% vs 75 of 270; 27.8%). Although the industry press sample was too small to allow a meaningful comparison, cardiothoracic surgery was represented in 3 of the 7 total articles (42.9%). Additionally, cardiothoracic surgeons appeared at nearly twice the rate in the medical press (99 of 854; 11.6%) compared with the lay press (16 of 270; 5.9%). Other physicians were represented in 43 of 270 (15.9%) of lay press articles and in 128 of 854 (15.0%) of medical press articles (Table 2).

**Table 2:** Specialty representation by press type

Specialty	Medical press (N = 852)	Lay press (N = 270)	Industry press (N = 7)
Cardiology	488 (57.1)	151 (55.9)	1 (14.3)
Interventional cardiology	202 (23.7)	75 (27.8)	0 (0)
Cardiothoracic surgery	99 (11.6)	16 (5.9)	3 (42.9)
Other physicians	128 (15.0)	43 (15.9)	1 (14.3)

Categorical data presented as n (%).

### Specialty Representation

**Figure 1:**
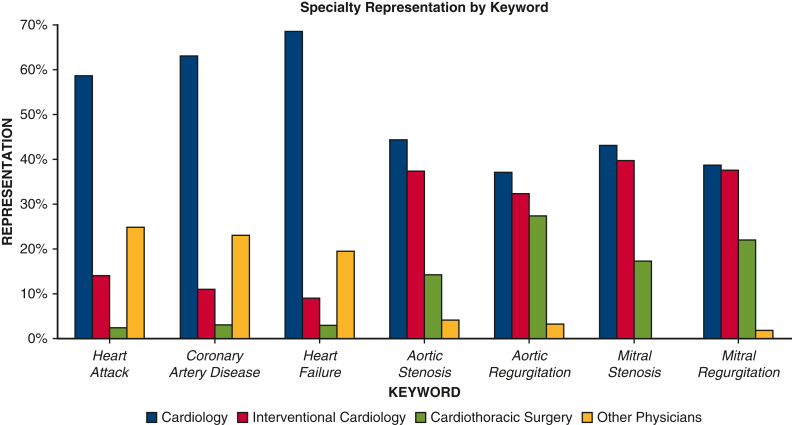
Specialty representation by keyword.

Table 3 and Figure 1 summarize the distribution of physician specialties represented across keywords. Among the 286 physicians contributing to “heart attack” articles, cardiologists constituted the majority (168 of 286; 58.7%), followed by other physicians (71 of 286; 24.8%). “Coronary artery disease” articles showed a similar pattern, with cardiologists the most represented (104 of 165; 63.0%) and other physicians contributing to a lesser extent (38 of 165; 23.0%). “Heart failure” articles were more frequently represented by cardiologists (144 of 210; 68.6%), with other physicians appearing second most frequently (41 of 210; 19.5%). Across the keywords “heart attack,” “coronary artery disease,” and “heart failure”—other physicians consistently had the second-highest level of representation after cardiologists. “Aortic stenosis” articles most often included cardiologists (109 of 246; 44.3%) and interventional cardiologists (92 of 246; 37.4%). “Aortic regurgitation” articles showed a similar distribution, with cardiologists (23 of 62; 37.1%) and interventional cardiologists (20 of 62; 32.3%) as the 2 most frequently represented specialties. “Mitral stenosis” articles also were contributed primarily by cardiologists (25 of 58; 43.1%) and interventional cardiologists (23 of 58; 39.7%). Similarly, “mitral regurgitation” articles featured the most visible representation from cardiologists (67 of 173; 38.7%) and interventional cardiologists (65 of 173; 37.6%). Cardiothoracic surgeons were least represented in articles on “heart attack,” “coronary artery disease,” and “heart failure” (7 of 286, 2.4%; 5 of 165, 3.0%; and 6 of 210, 2.9%, respectively). Within articles on valvular diseases—“aortic stenosis,” “aortic regurgitation,” “mitral stenosis,” and “mitral regurgitation”—cardiothoracic surgeon representation (35 of 246, 14.2%; 17 of 62, 27.4%; 10 of 58, 17.2%; 38 of 173, 21.9%, respectively) exceeded that of other physicians, although it remained lower than that of cardiology and interventional cardiology.

**Table 3:** Keyword representation by specialty

Keyword	Cardiology	Interventional cardiology	Cardiothoracic surgery	Other physicians	Total
Heart attack	168 (58.7)	40 (14.0)	7 (2.4)	71 (24.8)	286
Coronary artery disease	104 (63.0)	18 (10.9)	5 (3.0)	38 (23.0)	165
Heart failure	144 (68.6)	19 (9.0)	6 (2.9)	41 (19.5)	210
Aortic stenosis	109 (44.3)	92 (37.4)	35 (14.2)	10 (4.1)	246
Aortic regurgitation	23 (37.1)	20 (32.3)	17 (27.4)	2 (3.2)	62
Mitral stenosis	25 (43.1)	23 (39.7)	10 (17.2)	0 (0)	58
Mitral regurgitation	67 (38.7)	65 (37.6)	38 (22.0)	3 (1.7)	173
Total	640 (53.3)	277 (23.1)	118 (9.8)	165 (13.8)	1200

Categorical data presented as n (%).

Across all keywords, 470 cardiologists (73.4%) were academically affiliated, and 170 (26.6%) were non–academically affiliated (Table 4). Academically affiliated cardiologists were most frequently represented in “heart failure” articles (124 of 144; 86.1%), followed by “coronary artery disease” articles (82 of 104; 78.8%). Non–academically affiliated cardiologists were most represented in “aortic stenosis” articles (43 of 109; 39.4%), followed by “heart attack” articles (53 of 168; 31.5%). Overall, academically affiliated cardiologists had higher representation than non–academically affiliated cardiologists across all keywords. There were 88 (74.6%) academically affiliated cardiothoracic surgeons and 30 (25.4%) non–academically affiliated cardiothoracic surgeons. Academically affiliated cardiothoracic surgeons were most represented in “mitral stenosis” articles (9 of 10; 90.0%), followed by “mitral regurgitation” articles (31 of 38; 81.6%). Non–academically affiliated cardiothoracic surgeons were most represented in “heart attack” articles (4 of 7; 57.1%) (Table 4).

**Table 4:** Demographics in cardiology and cardiothoracic surgery

Characteristic	Heart attack	Coronary artery disease	Heart failure	Aortic stenosis	Aortic regurgitation	Mitral stenosis	Mitral regurgitation	Total
Cardiology								
Subspecialty								
Academic	115 (68.4)	82 (78.8)	124 (86.1)	66 (60.6)	17 (73.9)	18 (72)	48 (71.6)	470 (73.4)
Nonacademic	53 (31.5)	22 (21.2)	20 (13.9)	43 (39.4)	6 (26.1)	7 (28.0)	19 (28.4)	170 (26.6)
Sex								
Male	98 (58.3)	60 (57.7)	98 (68.1)	95 (87.2)	20 (87.0)	23 (96.0)	62 (92.5)	456 (71.3)
Female	70 (41.7)	44 (42.3)	46 (31.9)	14 (12.8)	3 (13.0)	2 (8.0)	5 (7.5)	184 (28.7)
Physician role								
Interviewee	151 (89.9)	96 (92.3)	133 (92.4)	104 (95.4)	23 (100)	25 (100)	65 (97.0)	597 (93.3)
Author	1 (0.6)	1 (1)	7 (4.9)	5 (4.6)	0 (0)	0 (0)	2 (3.0)	16 (2.5)
Reviewer	16 (9.5)	7 (6.7)	4 (2.8)	0 (0)	0 (0)	0 (0)	0 (0)	27 (4.2)
Cardiothoracic surgery								
Subspecialty								
Academic	3 (42.9)	4 (80)	4 (66.7)	26 (74.3)	11 (64.7)	9 (90)	31 (81.6)	88 (74.6)
Nonacademic	4 (57.1)	1 (20)	2 (33.3)	9 (25.7)	6 (35.3)	1 (10)	7 (18.4)	30 (25.4)
Sex								
Male	5 (71.4)	5 (100)	5 (83.3)	30 (85.7)	15 (88.2)	5 (50)	28 (73.7)	93 (78.8)
Female	2 (28.6)	0 (0)	1 (16.6)	5 (14.3)	2 (11.8)	5 (50)	10 (26.3)	25 (21.2)
Physician role								
Interviewee	7 (100)	5 (100)	6 (100)	34 (97.1)	15 (88.2)	10 (100)	38 (100)	115 (97.5)
Author	0 (0)	0 (0)	0 (0)	1 (2.9)	2 (11.8)	0 (0)	0 (0)	3 (2.5)
Reviewer	0 (0)	0 (0)	0 (0)	0 (0)	0 (0)	0 (0)	0 (0)	0 (0)

Categorical data presented as n (%).

### Gender and Physician Roles

In Table 3, among cardiologists, 456 (71.3%) were male and 184 (28.7%) were female, with male cardiologists showing higher representation across all keywords. Female cardiologist presence was highest in articles about “heart attack” (70 of 168; 41.7%), “coronary artery disease” (44 of 104; 42.3%), and “heart failure” (46 of 144; 31.9%). Among cardiothoracic surgeons, 93 (78.8%) were male and 25 (21.2%) were female. Male cardiothoracic surgeons had higher representation across all keywords except in articles on “mitral stenosis,” where both genders were represented equally (5 of 10; 50%). After “mitral stenosis,” female cardiothoracic surgeon presence was next highest in articles on “heart attack” (2 of 7; 28.6%) and “mitral regurgitation” (10 of 38; 26.3%).

Most physicians across all keywords served as interviewees, including 597 of 640 cardiologists (93.3%) and 115 of 118 cardiothoracic surgeons (97.5%), whereas 43 cardiologists (6.7%) and 3 cardiothoracic surgeons (2.5%) contributed as authors or reviewers (Table 4).

## Discussion

The implications of our findings for understanding patterns in online cardiovascular news ecosystems were explored across 5 domains: (1) specialty representation, (2) gender, (3) press type, (4) clinical practice, and (5) public health communication.

### Specialty Representation

Cardiologists were the most frequently represented specialty across all keywords, followed by interventional cardiologists, other physicians, and cardiothoracic surgeons. Although no study to date has systematically mapped clinician specialty representation in media coverage of common cardiovascular diseases, prior research suggests that journalists often rely on readily available clinicians and a limited pool of experts when reporting on acute or high-impact health stories.^7^, ^8^, ^9^ This tendency, along with other contributing factors, may help explain why cardiologists—recognized as central figures in cardiovascular disease and the heart team—are represented more frequently. Workforce differences also may contribute; according to the US Physician Workforce database, there are approximately 22,843 cardiologists and 4693 cardiothoracic surgeons—a ratio of approximately 5:1.^10^ Consequently, differences in media representation may reflect availability rather than true disparities, as cardiothoracic surgeons spend substantial time in the operating room and are less accessible. Cardiothoracic surgeons were less frequently represented in coverage of “coronary artery disease,” “heart failure,” and “heart attack,” which may reflect real-world care pathways, whereas they were more visible in articles on valvular diseases (“aortic stenosis,” “aortic regurgitation,” “mitral stenosis,” and “mitral regurgitation”). Interventional cardiologists showed a similar pattern, while other physicians were most visible in “heart attack” coverage. The greater presence of internal medicine and emergency medicine physicians in acute care scenarios (eg, “heart attack”) may align with media tendencies to highlight front-line clinicians associated with rapid diagnosis and triage.^1^ Media specialty representation also may reflect the keywords being reported (eg, “coronary artery disease” or “heart failure”) rather than systematic bias in the portrayal of cardiovascular care.

Moreover, academically affiliated cardiologists appeared most frequently in “heart failure” articles, reflecting the concentration of expertise in goal-directed medical therapy, mechanical circulatory support, and heart transplantation at academic centers. Academically affiliated cardiothoracic surgeons were most frequently represented in “mitral regurgitation” articles, consistent with the high-volume specialization in surgical mitral valve repair and transcatheter mitral edge-to-edge repair (TEER) at academic centers. Non–academically affiliated cardiologists were most frequently represented in “heart attack” and “aortic stenosis” articles, as percutaneous coronary intervention and TAVR are commonly performed in community hospitals. Similarly, non–academically affiliated cardiothoracic surgeons appeared most frequently in “aortic stenosis” articles, reflecting the incorporation of TAVR into community practice.

### Gender

Female physicians appeared less frequently than their male counterparts across all keywords, although their presence was highest among cardiologists featured in articles about “heart attack,” “coronary artery disease,” and “heart failure.” When considered alongside 2022 Association of American Medical Colleges data showing that cardiology and cardiothoracic surgery remain predominantly male domains (84.5% and 91.7%, respectively), these findings underscore a persistent gender gap in both the workforce and media.^11^ Women physicians are frequently less represented across many specialties and in media coverage, with fewer opportunities to serve as expert sources, while male clinicians dominate professional and public platforms as more frequently cited and positioned as authoritative lead commentators.^12^^,^^13^ Observed efforts to increase the visibility of women physicians may reflect awareness of gender inequities in cardiovascular medicine and initiatives to diversify journalistic expert sources.

### Press Type

Specialty visibility is shaped in part by structural differences across media sectors, illustrating how publication type can influence which medical specialties the public encounters.^14^ Industry outlets typically highlight procedural innovations (eg, TAVR, TEER), device development, and specialty-specific advances—topics that naturally emphasize interventional and surgical expertise. Indeed, cardiothoracic surgeons appeared more frequently in industry-focused publications, although the sample size was small. In contrast, lay media tend to emphasize patient narratives, broad explanations of disease, and content tailored to general audiences. Journalists often turn to first-contact or readily accessible clinicians who can translate complex medical information into clear, lay terms. This often includes family medicine, internal medicine, and emergency medicine physicians who support patients’ understanding of heart team referrals and decisions, as well as cardiologists directly involved in the relevant condition. Lay news outlets may simplify complex clinical topics, which may result in reduced visibility of specialties that are highly technical or procedure-driven.^1^ Therefore, the relatively lower presence of cardiothoracic surgeons in lay coverage may limit public exposure to surgical perspectives.

### Clinical Practice

Clinicians in highly visible acute-care roles, especially in front-line or procedure-oriented specialties, are often highlighted.^1^ For example, acute coronary syndrome is typically diagnosed by emergency medicine physicians and subsequently managed by interventional cardiologists through percutaneous coronary intervention. Similarly, “coronary artery disease” is initially managed with lifestyle modifications and antiplatelet and lipid-lowering agents by generalists, and “heart failure” is typically treated with goal-directed medical therapy by cardiologists.^15^^,^^16^ Less frequent surgical presence in media discussions of “coronary artery disease” and “heart failure” may mirror these disorders’ predominantly medical management and reflect typical clinical management patterns. Cardiothoracic surgeons provide first-line treatment for valvular diseases, including “aortic stenosis,” “aortic regurgitation,” “mitral stenosis,” and “mitral regurgitation,” which may contribute to their relatively strong representation in these conditions. As procedures such as TAVR and TEER continue to increase in volume, cardiothoracic surgeons remain actively involved in these interventions.^17^ Coverage of aortic stenosis shows a greater representation of cardiologists and interventional cardiologists, likely reflecting the rapid adoption of TAVR, the procedural volume of which now exceeds that of its surgical counterpart.^18^ As such, the growth of interventional procedures has shifted public and journalistic attention toward interventional cardiology. These patterns suggest that specialty representation reflects both media sourcing norms and clinical practice trends, including disease-specific care pathways and initial points of patient contact.

### Public Health Communication

With nearly three-quarters of adults using online sources for health information, patients are increasingly relying on the internet as a first-line resource.^19^ Although social media platforms provide immediate access to vast amounts of information, traditional online news media remain perceived as more credible for medical topics, particularly among older adults and individuals seeking evidence-based guidance.^20^, ^21^, ^22^ We show how cardiovascular news reaches the public, with physicians—most often cardiologists—playing a key role in preserving clinical nuance, interpreting evidence accurately, and countering public health misinformation. Journalistic framing occasionally emphasizes the most visible perspectives. This was evident in coverage of the 2023 American College of Cardiology/American Heart Association Chronic Coronary Disease Guidelines, where perspectives from the Society of Thoracic Surgeons and the American Association for Thoracic Surgery were featured less prominently and did not consistently shape dominant media narratives.^23^^,^^24^ These selective emphases can result in the public receiving an incomplete understanding of the evolving evidence and the guideline development process. For this reason, interdisciplinary communication incorporating multiple clinical perspectives may help balance single-specialty viewpoints. Partnerships among professional societies and other stakeholders can give journalists access to a wider pool of experts, foster collaborative communication, and enhance public understanding of cardiovascular disease.

### Limitations

Differences in media representation may reflect cardiologists’ greater availability and the keywords being reported rather than true disparities with cardiothoracic surgeons or systematic bias in the portrayal of cardiovascular care. The present analysis relied on online sources of variable quality across specialties, and the use of diagnostic keywords might have disproportionately captured cardiology-related discourse while underrepresenting contexts in which cardiothoracic surgeons are featured more prominently, thereby shaping specialty visibility. The limited keyword set could have excluded relevant data, affecting the validity and generalizability of findings, which precluded formal hypothesis testing. The 1-year study window limits assessment of temporal trends and whether representation patterns are stable or evolving. Observed patterns also may reflect disease-specific care pathways and points of first clinical contact rather than purely preferential journalistic selection. Despite efforts to reduce bias from Google search algorithms, their complex and opaque nature might have influenced article inclusion, particularly in lay media outlets, while amplification bias from Google News relevance rankings may have favored high-visibility outlets (eg, academic cardiology departments) and commonly cited specialties (eg, cardiology) regardless of journalistic intent. Finally, reliance on Google News without assessing interrater reliability may have introduced bias and reduced reproducibility.

## Conclusions

Cardiologists appeared in more than one-half of online articles covering common cardiovascular diseases. Cardiothoracic surgeons were represented less frequently in coverage of “heart attack,” “coronary artery disease,” and “heart failure” but appeared more often in articles on valvular disease. For “heart attack,” “coronary artery disease,” and “heart failure,” other physicians consistently had the second-highest level of representation after cardiologists. Female physicians were represented less frequently than their male counterparts. This study does not provide prescriptive guidance for journalistic engagement or directly evaluate heart team equity but instead characterizes contemporary patterns of physician representation in online cardiovascular news through a descriptive analysis.

## Conflict of Interest Statement

Dr Bavaria reported consulting for Edwards Lifesciences, Terumo Aortic, Abbott, Medtronic, and Artivion. All other authors reported no conflicts of interest.

The *Journal* policy requires editors and reviewers to disclose conflicts of interest and to decline handling or reviewing manuscripts for which they may have a conflict of interest. The editors and reviewers of this article have no conflicts of interest.
